# Regional Node Distribution in Papillary Thyroid Cancer with Microscopic Metastasis

**DOI:** 10.1155/2018/1718284

**Published:** 2018-11-01

**Authors:** Luis-Mauricio Hurtado-López, Alejandro Ordoñez-Rueda, Felipe-Rafael Zaldivar-Ramírez, Erich Basurto-Kuba

**Affiliations:** Thyroid Clinic, General Surgery Service, Hospital General de México, Mexico City, Mexico

## Abstract

**Background:**

Optimal neck lymphadenectomy in patients with papillary thyroid cancer (PTC) and microscopic lymph node metastasis needs to be defined in order to aid surgeons in their decision about the best way to proceed in these cases.

**Methods:**

Patients who underwent total thyroidectomy and lymphadenectomy at levels IIa to VI were divided into two groups: Group 1 (G1) with macroscopic metastasis detected before surgery and Group 2 (G2) with microscopic metastasis detected in sentinel node during surgery. Odds ratio (OR) was computed for age, sex, tumor size, multicentricity, capsular invasion, vascular/lymphatic permeation, and nodes with metastasis.

**Results:**

Primary tumor size was (G1 versus G2, respectively) 3.8 cm versus 1.98 cm (P<0.001); only lymphatic permeation was correlated to an increase in metastasis in lymph nodes 65.4% versus 25% (OR=5.6, p<0.001); metastatic frequency by region was IIa 18.5% versus 1.5%, III 24.3% versus 9.9%, IV 17.4% versus 18.1%, and VI 25.9% versus 71,2%. Metastasis to level V was found only in G1.

**Conclusion:**

Selective lymphadenectomy at levels III, IV, and VI is optimal for PTC patients without preoperative evidence of lymph node disease, but who present with lymph node microscopic metastasis in an intraoperative assessment.

## 1. Introduction

The presence of papillary thyroid cancer (PTC) in lymph node metastasis can only be determined at the time of diagnosis with a frequency of 30% to 40% [1–8]. Regional metastatic PTC disease has been identified as an independent risk factor for recurrence, including morbidity under certain conditions [9, 10]. For example, when substratification indicates that six or more nodes are affected, this can determine higher mortality rates [11]. Recurrence increases with the number of metastatic nodes and the degree of metastasis, as well as with extracapsular infiltration and age [12].

A lymphadenectomy in PTC is only indicated when lymph node metastasis has been established with certainty [13]. Two clinical scenarios are possible: the first occurs in the preoperative assessment, when large lymph node metastases are found by palpation or ultrasound (US), and fine-needle aspiration biopsy (FNAB) is performed and then evaluated with cytology and thyroglobulin assay to corroborate the presence of PTC in the lymph nodes [14–16]. The second scenario is for patients without preoperative evidence of lymph node disease (cN0), but who present with lymph node microscopic metastasis in an intraoperative assessment, by a sentinel lymph node [17–20].

In each of the above-mentioned conditions, the surgeon should perform a lymphadenectomy. However, in patients with microscopic metastasis only, many surgeons choose to omit the lymphadenectomy and instead keep the microscopic carcinoma under observation. Of those who do choose to perform a lymphadenectomy, the majority typically perform a functional radical neck dissection from levels IIa to V, as well as level VI. However, an important surgical principle is to perform a lymphadenectomy only at the affected levels, in order to avoid unnecessary morbidity [5, 13, 21–23]. In this sense, we believe that a functional radical neck lymphadenectomy is not the best option in the case of lymphatic microcarcinoma metastasis.

Analyses of recurrence and mortality related to the presence of PTC regional metastatic disease clearly indicate a correlation between the number and size of metastatic nodes [9–12]. Large lymph node metastasis has a strong prognostic relationship to carcinoma recurrence, so when palpable lymph node metastases are detected at any level, a lymphadenectomy at levels IIa to VI is justified [5, 21–24]. However, when a metastatic node is detected through a sentinel lymph node, we are dealing with microscopic metastases, and it is then unclear if the same extended lymphadenectomy should be performed, particularly considering the low probability of recurrence, as the tumors are very small [12]. Nevertheless, PTC frequently metastasizes to level VI (42%) [13] and recurrence in lateral lymph nodes after a total thyroidectomy and Radioactive iodine (RAI) therapy of these cases is present in 4.5% to 24.5% [25–27], which is likely due to the noneffective rate of RAI therapy [28]. While this recurrence may not be fatal immediately, it is troubling for both physicians and patients because of the resultant need for additional surgery or RAI.

The aim of the present study is to identify the regional distribution pattern of PTC in order to recommend the optimal extension of lymphadenectomy levels when microscopic metastasis is present in these patients.

## 2. Materials and Methods

We performed a prospective, observational, comparative study in two groups of patients with well differentiated papillary thyroid cancer, carried out for 24 months at the Thyroid Clinic of the Hospital General de Mexico.

G1 was comprised of patients with PTC and regional macroscopic metastatic activity, demonstrated by lymph node palpation, US, and preoperative FNAB which confirmed regional metastatic activity. This group of patients underwent a total thyroidectomy (TT) and lymphadenectomy from lymphatic IIa to VI. G2 was comprised of PTC patients with neither a palpable lymph node, nor a node demonstrated via US, but for whom we did find a sentinel lymph node during surgery (detected by patent blue), which was positive for microscopic metastatic activity (which implies lymph node equal to or less than 1 cm in which only microscopic evidence of tumor activity exists). These patients also underwent a TT and omolateral lymphadenectomy from lymphatic levels IIa to VI.

The lymphadenectomy (lymphatic levels IIa to VI) was performed following the guidelines of the American Thyroid Association consensus review, with a statement regarding the anatomy, terminology, and rationale for lateral neck dissection in differentiated thyroid cancer [29], as well as the consensus statement regarding terminology and classification of central neck dissection for thyroid cancer [30]. All resected lymphatic levels were separated by the surgeon in the surgical field and sent separately for pathological assessment. The sentinel lymph node was determined by injecting 0.5 ml of patent blue (Isosulfan Blue Vital Dye; Lymphazurin 1%, Hirsch Industries, Richmond, VA, USA) into the primary tumor, following its visual dissemination until finding the blue-stained node after one minute, resection, and intraoperative histopathological identification of metastasis.

Variables evaluated were age, gender, size of the primary tumor, presence of multicentricity, capsular invasion, vascular permeation, lymphatic permeation in the primary tumor, as well as the metastatic frequency at each of the resected lymphatic levels, and rate of main surgical complications (hypoparathyroidism, recurrent, vagus, accessory spinal, marginal, hypoglossal, phrenic nerves, sympathetic chain, brachial plexus, and thoracic duct injury)

The study was performed at the General Hospital of Mexico, a tertiary level hospital, which has several research, ethics, and legal committees. Patients were informed of all surgical procedure protocols and their possible consequences upon their admittance, and a written consent to publish the findings was obtained in accordance with the institutional review board.

Data analysis was done using IBM SPSS Statistics 20 © software. Statistical analysis was performed with central tendency measures, and comparisons were made using Chi-square (X^2^), Fisher exact test, and Student's t-test at a significance level of P < 0.05 and OR, which corresponds to a higher probability by using the following formula: probability = (OR/OR+1) × 100.

## 3. Results

During the studied period, there were 635 thyroidectomy patients, of these 223 were due to well differentiated papillary thyroid cancer, of which 52 had the characteristics to form G1 and 171 patients with neither a palpable lymph node, nor a node demonstrated via US with characteristics of regional metastasis; from these 171 in 62 patients we perform a sentinel lymph node during surgery (detected by patent blue) and the G2 was formed.

G1 was composed of 40 women and 12 men, with an average age of 42 years (range of 17 to 67 years); G2 was composed of 36 women and 4 men, with an average age 39.7 years (range of 19 to 58 years). Comparison of these groups made them homogeneous and therefore statistically comparable. G2 was formed from an initial population of 62 patients who underwent sentinel lymph node evaluation, and of these, 40 (64.5%) were positive for lymph node microscopic PTC metastasis.

Size of the primary tumor for G1 was 3.8 cm (range 2 cm to 4 cm) and for G2 it was 1.98 cm (range 1 cm to 2.5 cm); the comparison with Student's t-test yielded a P = 0.0001.

G1 presented 12 (23%) cases of multicentricity, whereas G2 presented only 2 cases (5%). The two-tailed Fisher exact test yielded P = 0.0196

Capsular invasion of the primary tumor was found in 32 (61.5%) cases of G1 and 18 (45%) cases of G2. The two-tailed Chi-squared test yielded P = 0.1144.

Vascular permeation in the primary tumor was present in 24 (46.15%) cases of G1, and 14 (35%) cases of G2. The two-tailed Chi-squared test yielded P = 0.2814.

Assessment of lymphatic invasion in the primary tumor was in 34 (65.38%) cases of G1 and in 10 (25%) cases of G2. The two-tailed Chi-squared test yielded p = 0.0001, OR = 5.6667 (95%CI, 2.2679 to 14.1592). Probability was 84.8%.

Regarding G2 patients, of the 40 with metastasis in the sentinel node, the most frequent localizations were level VI with 32 (80%) cases (of these, 16 corresponded to paratracheal node and 16 to pretracheal node). These 32 patients with a positive sentinel node at level VI had at least one additional metastatic node at the lateral level. The remaining 8 sentinel node patients corresponded to level IV only.

The 16 level VI paratracheal nodes and 8 level IV nodes corresponded to tumors in the upper half of the affected thyroid lobe; however, the 16 level VI pretracheal nodes corresponded to primary tumors in the lower half of the affected lobe.

In G1, 1170 nodes were obtained, of which 378 were positive for macro- and microscopic metastatic activity. In G2, 694 nodes were obtained, of which 132 were positive for microscopic metastatic activity. The distribution and percentage of positive nodes, according to the studied level per group, are depicted in Table 1.

No patient from both groups presented complications related to lymphadenectomy

## 4. Discussion

This study clearly demonstrates that, in the presence of a sentinel lymph node with macroscopic metastases, there is at least one additional level with metastatic activity in the cervical lymph nodes, with level VI, and lateral levels III and IV being the most frequently affected. There were no cases of metastasis at level V; therefore, this level can be safely excluded from lymphadenectomies in this kind of patient. This study also demonstrates that the presence of lymphatic permeation in the primary tumor correlates to an 84.4% probability of lymphatic metastasis.

This allows us to use these results in the specific clinical scenario under evaluation: a papillary thyroid cancer patient without preoperative evidence of lymphatic node metastasis, but with interoperative findings of lymph node micrometastasis. Yet, it also allows us to use the information for decisions regarding other hypothetical, but frequent, clinical scenarios, such as when a total thyroidectomy without lymphadenectomy is performed for PTC and lymphatic permeation in the primary tumor is found or when a total thyroidectomy is performed along with routine dissection of the central compartment (level VI) and the final histological report indicates lymphatic permeation and at least one lymphatic micrometastasis. In these three clinical scenarios, what procedure should be followed? Should we wait for a recurrence or for the persistence to manifest? Or should we perform selective lymphadenectomy of levels III, IV, and VI? In reality, the answer will depend on the surgeon's knowledge and understanding of the malignant neoplasm.

PTC should be studied from a longitudinal perspective, as time takes on a fundamental significance with this type of tumor which, as a result of its nonaggressive biological behavior, allows for multiple analyses, and which, if visualized transversally, can lead us to incorrect conclusions; it is important to keep in mind that we are still referring to the same malignant neoplasm, only in different moments of time. While the presence of large lymphatic metastases clearly influences reoccurrence and morbidity/ survival rate, this might not appear to be the case when the metastases are microscopic. However, “microscopic” only means that the disease is in a different moment in time, but it is still the same disease, and so, one can decide at which moment to intervene in the natural evolution of the disease, be it at a later stage (large lymph nodes), or earlier stage (micrometastasis).

This concept has been shown historically: we can compare Mazzaferi's first study (1977) [31] and latest study (2001) [32] to understand clearly how time is the main player, as it demonstrates not only the natural evolution of the disease, but also clearly infers the therapeutic concept that this study recommends. The same is true when considering the initial study done by Ito [33] where microcarcinomas were left under observation; 5 and 10 years later, 4.55% and 8% had progressed with 15% requiring surgical intervention [34]. If we wait for a new study every five years, surely this percentage will continue to increase. Similarly, if we only analyze with transversal thought, we run the risk of reaching conclusions that would make it seem that time is of little importance to metastatic lymph nodes.

Another essential point in understanding PTC is that the 5-year timeframe that we use to evaluate diverse cancers in the human body and measure disease-free survival, recurrence, and global survival rates does not apply to PTC due to its slow evolution, and therefore, this period of evaluation should be extended to at least 20 years. It is also important to extend this period, given that that the majority of patients currently being diagnosed and treated for PTC are young and without a doubt have the possibility of living for 20 or many more years.

Taking into consideration the above, the information generated from this study can be used in two ways: the first is for the scenario under review, when a sentinel lymphatic node is positive for microscopic metastasis, in which it would be sufficient to perform a selective lymphadenectomy of levels III, IV, and VI, in addition to the local surgery, rather than a functional radical neck lymphadenectomy. The second scenario is when no lymphadenectomy has been performed, but the primary tumor has lymphatic permeation. Even with a therapeutic adjuvant dose of RAI, the surgeon should maintain close contact with the evolution of the patient, considering that up to 12% of lymphatic microscopic metastases do not capture iodine and hence can manifest later or even be confused with a recurrence when, in fact, it was a persistent tumor.

It is important to mention that, in the present study, microscopic metastatic activity in G2 at the IIa level represented only 1.5% of metastases. Thus, we recommend that it be excluded from lymphadenectomy. However, in the strictest sense, level IIa could also be included in this situation, at the criteria of the surgeon.

Thus, all surgeons must decide, once they have understood the natural evolution of the disease, when to intervene, taking into consideration the age of the patient, as well as the educational level and characteristics specific to the population being treated, as some will choose to leave the disease to evolve freely and await further changes before initiating treatment, while others will decide to intervene in the natural evolution at an earlier stage. An attitude of deep professionalism would be for the surgeon to inform the patient, at the onset, of all possible outcomes of this disease, so that the patient and doctor may decide together, what actions to take.

We conclude that there is a difference in the distribution of lymph node metastasis between the two clinical conditions studied. In this sense, we found that in papillary thyroid cancer patients without metastatic node detection in the preoperative assessment, but with microscopic lymphatic metastasis detected interoperatively, selective lymphadenectomy at levels III, IV, and VI is optimal and should be preferred over functional radical neck lymphadenectomy and, in the case of younger patients, over monitoring for changes.

## Figures and Tables

**Table 1:** Distribution and percentage of positive nodes.

Cervical lymph node level	Number of positive lymph nodes	
	G1 n(%)	G2 n(%)
IIa	70(18.52)	2(1.51)
III	92(24.34)	12(9.09)
IV	66(17.46)	24(18.19)
Va	12(3.17)	0
Vb	40(10.58)	0
VI	98(25.93)	94(71.21)

## Data Availability

The data used to support the findings of this study are available from the corresponding author upon request.
